# Genomic sequence of three hydrocarbonoclastic pseudomonadaceae strains isolated from marine sediments of the port of Rosarito, Baja California, Mexico

**DOI:** 10.1128/mra.00335-24

**Published:** 2024-06-25

**Authors:** Sofía Millán-López, Juan M. Zurita-Artaloitia, Hortencia Silva-Jiménez, Liliana Pardo-López, Alejandro Sanchez-Flores

**Affiliations:** 1 Departamento de Microbiología Molecular, Instituto de Biotecnología, Universidad Nacional Autónoma de México, Cuernavaca, Morelos, Mexico; 2 Instituto de Investigaciones Oceanológicas, Universidad Autónoma de Baja California, Ensenada, Baja California, México; 3 Unidad Universitaria de Secuenciación Masiva y Bioinformática, Instituto de Biotecnología, Universidad Nacional Autónoma de México, Cuernavaca, Morelos, México; Montana State University, Bozeman, Montana, USA

**Keywords:** marine bacteria, hydrocarbon degradation, bioremediation, genomics, comparative genomics

## Abstract

We report the draft genome sequence of three marine bacteria belonging to *Pseudomonas* and *Stutzerimonas* genera, with hydrocarbonoclastic metabolism for oil and monoaromatic hydrocarbon degradation. The genomic information of these organisms contributes to the knowledge of natural and polluted marine environments with ubiquitous presence of hydrocarbons as a selective pressure.

## ANNOUNCEMENT

**Fig 1:**
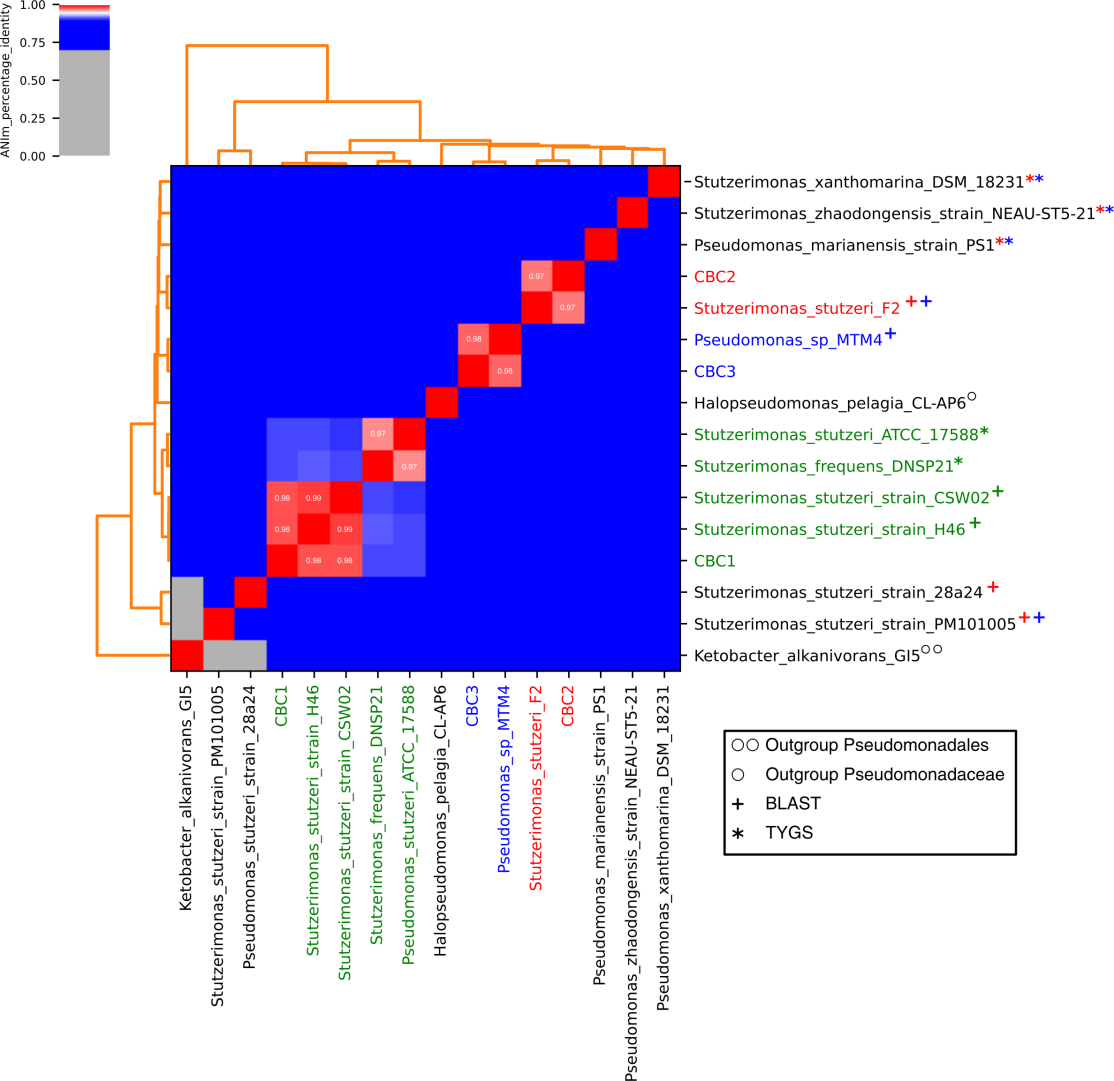
ANIm percentage identity heatmap of the three isolates against their two closest related sequences and reference genomes from *Pseudomonas* and *Stutzerimonas*. Genomes from strains corresponding to Pseudomonadaceae (*Halopseudomonas*, marked with a ੦) and Pseudomonadales (*Ketobacter*, marked with a ੦੦) were used as outgroups. The strain CBC2 is within the cluster highlighted with red, while CBC3 and CBC1 are blue and green, respectively. The squares with an ANI% between 0.96 and 1 are annotated. The close related sequences obtained from the TYGS are marked with an “*” and the ones obtained from the blastn are marked with a “+.”

Oceanic oil presence can be the product of anthropogenic activities or could be ubiquitous in some regions worldwide. It acts as a selective pressure for microorganisms to evolve mechanisms to cope with it and eventually use it as an energy source. In some cases, understanding these mechanisms leads to novel solutions for bioremediation of polluted environments. In this study, we report the draft genome sequences of three marine bacteria pure isolates from marine sediments (from Rosarito Port, Baja California, Mexico) as described in detail previously (1). Additionally, the isolates showed the capacity to grow in the presence of Bacab alpha oil (0.01%) or phenol (0.05%) as sole carbon source in a Bushnell Haas broth (BH) medium. From each isolate, genomic DNA was extracted using the Quick-gDNA Miniprep Kit (Zymo Research) and sequencing libraries were prepared with the Illumina DNA Prep library kit (Illumina, Inc.). The Illumina MiSeq platform with a 600 cycle kit in a 2 × 300 bp configuration was used for sequencing. The quality read assessment was performed using FASTQC (v.0.12.1) (2) and genome assembly was done with the Discovar Denovo (v52488) (3). The assembly quality test was made with Quast (v5.2.0) (4). Subsequently, the gene prediction and annotation were made using Bakta (v1.8.2) with the full database option (5). The completeness and contamination test were done with CheckM (v1.2.2) (6). The assembly, annotation, and quality results for the three isolates are reported in Table 1. The average nucleotide identity (ANI) test was conducted with the software PyAni (v0.2.12) (7), using MUMmer 3.0 (8). The ANI results can be consulted in Fig. 1. Default parameters were used for all software.

**TABLE 1:** Genomic features of the assembly, annotation, and quality analysis of the three strains

Genome features (proposed species)	CBC1 (*Stutzerimonas stutzeri*)	CBC2 (*Stutzerimonas stutzeri*)	CBC3 (*Pseudomonas* sp.)
Isolation coordinates	N 32°21′53.424″ O 117°4′42.42″	N 32°21′53.424″ O 117°4′42.42″	N 32°22′12.205″ O 117°4′ 42.531″
Assembly size (bp)	4,605,172	4,682,747	4,377,712
Sequencing depth (×)	695	732	795
G + C content (%)	63.86	62.65	60.85
No. of paired reads (2 × 301 bp)	2,657,625	2,847,413	2,889,613
No. of scaffolds	134	34	33
N50 (bp)	163,400	597,360	1,163,705
L50	8	3	2
Completeness	99.66	99.8	99.8
Contamination	0.14	0.14	0.14
No. of CDSs	4,250	4,184	3,973
No. of tRNAs	56	52	53
No. of rRNAs	9	8	9
GenBank accession no.	JAXBVD000000000	JBANEN000000000	JBANEO000000000
SRA accession no.	SRR28227299	SRR28227297	SRR28227379

Initially, comparative and distance analyses were performed by complete genome pairwise comparison using the Type Strain Genome Server (TYGS) v387 (9). The CBC1 strain was classified as *Stutzerimonas stutzeri* but for CBC2 and CBC3 strains, no species classification was achieved. Therefore, we performed an NCBI blastn search (10) using the largest contig from each genome and the genomes from the top three BLAST hits were used for an ANI test, where the type genomes suggested by TYGS were also included. Our analysis showed that CBC2 and CBC3 can be classified as *S. stutzeri* (97% ANI) and *Pseudomonas* sp. (98% ANI), respectively. The CBC1 isolate was confirmed as *S. stutzeri* (98% ANI). Considering the ANI values (above 97%), we assigned the same species as their closest related organism, except for CBC3, where only genus classification was available (Table 1). Despite CBC1 and CBC2 being characterized as the same species, they are not closely related. Conversely, the distance between CBC2 and CBC3 genomes is shorter, raising the question about the relation between genomic information and taxonomy. Interestingly, several predicted proteins belong to metabolic pathways related to hydrocarbon and monoaromatic compounds’ degradation, which is consistent with the carbon sources used for growth. Further investigation with a pangenomic approach will be conducted to understand the hydrocarbonoclastic metabolism evolution.

## Data Availability

Data availability for Whole Genome Shotgun projects have been deposited in GenBank under the accession numbers JAXBVD000000000, JBANEN000000000, and JBANEO000000000 for isolate CBC1, CBC2, and CBC3, respectively. In the same order, the Sequence Read Archive (SRA) accession numbers are SRR28227299, SRR28227297, and SRR28227379. The versions described in this paper are the first versions.
